# Chemical Composition, Antifungal and Insecticidal Activities of the Essential Oils from Tunisian *Clinopodium nepeta* subsp. *nepeta* and *Clinopodium nepeta* subsp. *glandulosum*

**DOI:** 10.3390/molecules25092137

**Published:** 2020-05-02

**Authors:** Haïfa Debbabi, Ridha El Mokni, Ikbal Chaieb, Simona Nardoni, Filippo Maggi, Giovanni Caprioli, Saoussen Hammami

**Affiliations:** 1 Research Unit 13ES63, Applied Chemistry and Environment, Faculty of Sciences of Monastir, University of Monastir, 5000 Monastir, Tunisia; debbabi_haifa10@hotmail.com (H.D.); h_saoussen@yahoo.fr (S.H.); 2 Department Pharmaceutical Sciences “A”, Laboratory of Botany, Cryptogamy and Plant Biology, Faculty of Pharmacy of Monastir BP 207, Avenue Avicenna, University of Monastir, 5000 Monastir, Tunisia; riridah@yahoo.fr; 3 Department of Silvo-Pastoral Resources, Laboratory of Research in Silvo-Pastoral Resources, Silvo-Pastoral Institute of Tabarka, BP. 345, University of Jendouba, Tabarka 8110, Tunisia; 4 IRESA, Laboratory of Forest Ecology, I.N.R.G.R.E.F, BP N°10, Ariana 2080, Tunisia; 5 Research unit UR13AGR09, Regional Center for Research in Horticulture and Organic Agriculture, Chott Mariem, University of Sousse, TN-4042 Sousse, Tunisia; ikbal_c@yahoo.fr; 6 Department of Veterinary Sciences, Università degli Studi di Pisa, 56124 Pisa, Italy; simona.nardoni@unipi.it; 7 School of Pharmacy, University of Camerino, 62032 Camerino, Italy; filippo.maggi@unicam.it

**Keywords:** Lamiaceae, *Clinopodium nepeta* subsp. *nepeta*, *Clinopodium nepeta* subsp. *glandulosum*, essential oils, chemical variability, biological activities

## Abstract

The present investigation was focused on the study of the chemical composition variability and biological activities of the essential oils from *Clinopodium nepeta* subsp. *nepeta* and subsp. *glandulosum*. Essential oils extraction was performed using hydrodistillation and the separation of the constituents was carried out by gas chromatography coupled with mass spectrometry (GC-MS). Antifungal activities were tested against *Aspergillus flavus, Aspergillus terreus*, *Microsporum canis*, *Microsporum gypseum*, *Trichophyton mentagrophytes,* and *Candida albicans*. Toxicity and repellency were evaluated against the stored product pests *Tribolium confusum* and *Sitophilus zeamais*. Both essential oils were characterized by a high content of oxygenated monoterpenes. Piperitone ranks first in the subspecies *nepeta* and piperitenone oxide is the dominant constituent in the subspecies *glandulosum*. All tested samples displayed noteworthy antifungal properties, with the highest activity observed for the essential oil of *C. nepeta* subsp. *glandulosum*, collected in Béni-M’tir, against *T. mentagrophytes* (MIC = 40 µg/mL). The essential oil samples of *C. nepeta* subsp. *glandulosum* were strongly repellent to the insect species (PR > 80%, after 2h) and highly toxic to *S. zeamais* reaching 97.5%–100% mortality after 24 h of exposure. In conclusion, this study showed considerable intra-specific changes in the quality of *C. nepeta* essential oils, which is reflected in different rates of antifungal and insecticidal activity.

## 1. Introduction

All across the globe, environmental problems such as soil and water pollution and food contamination are continuously increasing, inducing many disasters and human tragedies. The excessive and indiscriminate use of available pesticides to control the losses of stored crops and to reduce insect-borne diseases as malaria, filaria and trypanosomiasis induced disturbances in ecosystem functioning [1,2]. Moreover, synthetic fungicides and fungal-drugs utilization to treat environmental and animal molds are not in a lesser class, when we talk about the negative effects and the appearance of fungicide-resistant pathogens [3,4]. Therefore, there is an increasing concern to search for new highly selective and eco-friendly alternatives of beneficial pest control materials to feed the growing human population in a healthy environment. Medicinal plants produce an arsenal of chemical compounds that alleviate various illnesses and rebalance human health. Much attention has been focused on the study of plant extracts and essential oils due to their pivotal role as a source of phototherapeutics widely used to fight infectious diseases [5,6]. Natural insecticides, fungicides and herbicides with promising effects have the properties to supplant or replace synthetic organic pesticides and therefore to avoid environmental contamination. Hence, various applications of essential oils acting as safe fungicidal agents against a large number of molds and as environmentally friendly insecticides have been reported [7,8]. It is noteworthy that the biosynthesis and accumulation of phytochemicals by medicinal herbs are influenced by environmental circumstances including temperature, climate, light and the region altitude [9]. According to the harvesting time and the environment in which they are found, the same plant species or subspecies may present different chemical compositions of essential oils [10]. Importantly, the nature of the produced secondary metabolites and essential oils influences the mechanisms of actions and determines the medicinal and economic utility of various plants. In this context, differences in essential oils productivity, including an increase or decrease in the yields, and variation of the chemical composition depending on harvesting phase (vegetative, pre-flowering, flowering and fruiting), as well as the plant geographical origin have been previously investigated [11,12].

The genus *Clinopodium* (Lamiaceae family) consists of 135 perennial herbs, most of them being rich sources of essential oils, distributed widely in Southern Europe, western Asia and all around the Mediterranean area [13]. Many preclinical studies have demonstrated the inhibitory effects of *Clinopodium* essential oils towards various bacterial and fungal strains. Their insecticidal properties have also been reported [14,15,16,17]. Among various *Clinopodium* species, *Clinopodium nepeta* L. Kuntze (Syn. *Calamintha nepeta* L. Kuntze) is apolymorphic and a fragrant plant that has been used traditionally around the world as an important antispasmodic, diaphoretic, stimulant, and tonic medicinal herb; it is also considered as a mint-like spice and is used in various culinary recipes [18,19]. A literature survey of the chemical investigation of essential oils produced by *C. nepeta* revealed the high content in oxygenated monoterpenes. Most of the phytochemical and biological studies on *C. nepeta* did not concern the level of subspecies [20]. However, some authors take this parameter into consideration during analysis. As a consequence, the presence of remarkable variations among the major constituents of various subspecies was distinguished [21]. Their biological features have also been well-confirmed [22,23]. Thus, antimicrobial, anti-*Candida*, antioxidant and insecticidal activities of *C. nepeta* subsp. *glandulosum* (Req.) Govaerts essential oils from Montenegro, Turkey, Italy, and Croatia have been reported [24,25,26,27,28].

Since antiquity, the essential oils from *C. nepeta* subsp. *nepeta* leaves have been used as a fragrance and insect repellent [29]. The essential oils from the subspecies collected in Portugal, Serbia and Italy have been characterized for antifungal, antiproliferative, antioxidant, and antimicrobial effects [30,31,32,33]. In Tunisia only two subspecies, namely *C. nepeta* subsp. *nepeta* and *C. n.* subsp. *glandulosum* have been reported up to now; they have a very attractive smell and are often visited by insect pollinators. As far as we know, nothing is reported on the chemical profile and biological efficacy of their essential oils.

The aim of this paper was to compare the phytochemical profile and to test the biological efficiency of the essential oils extracted from *C. nepeta* subsp. *nepeta* and *C. nepeta* subsp. *glandulosum* growing in different localities in North and North-western Tunisia (Table 1). A comprehensive evaluation of the antifungal and insecticidal activities was investigated considering the chemical variability depending on the subspecies and the geographical origin. The main objective of this study was to search for environmentally friendly insecticides and antifungals that are readily biodegradable, with minimal toxic effects on health and environment and which can be marketed at premium prices. 

## 2. Results

### 2.1. Chemical Profiles of Essential Oils

The hydrodistillation of dry plant materials yielded 1.21%, 0.93% and 0.84% of essential oils, for *Clinopodium nepeta* subsp. *nepeta* (CNN), *Clinopodium nepeta* subsp. *glandulosum* from Béni-M’tir (CNG_1_), and *Clinopodium nepeta* subsp. *glandulosum* from Bizerta (CNG_2_), respectively. The percent occurrence of the oil phytochemicals, elucidated through GC-MS, is summarized in Table 2. 

As shown, 24, 40 and 42 components were identified and quantified in CNN, CNG_1_ and CNG_2_ essential oils accounting for 88.0%, 91.2% and 82.4% of the total compositions, respectively. Oxygenated monoterpenes constituted the main groups in CNN (82.0%), CNG_1_ (77.6%) and CNG_2_ (73.4%) essential oils of the selected Lamiaceae plants. It is worth noting that piperitone oxide and piperitenone oxide were identified as the most abundant components in the volatile oils of CNN (51.7% and 23.4%), CNG_1_ (23.5% and 39.3%) and CNG_2_ (16.3% and 27.8%). While the oxygenated monoterpene piperitone was found in high levels (19.5%) only in CNG_2_ (Table 2).

### 2.2. In Vitro Antifungal Activity of Essential Oils 

The anti-fungal activity of the selected essential oils was screened against potentially pathogenic fungi in humans and animals, namely *A. flavus, A. terreus, C. albicans, M. canis, M. gypseum,* and *T. mentagrophytes*, as shown in Table 3. 

In general terms, the tested fungal isolates showed a variable degree of sensitivity patterns to the selected essential oils. The Minimal Inhibitory Concentrations (MIC) ranged from 0.04 to 2 mg·mL^−1^. It was found that *A. terreus* and *M. canis* molds were moderately sensitive to all tested samples exhibiting a MIC value of 0.4 mg·mL^−1^. It is relevant to note that the CNG_1_ essential oil was considerably able to inhibit the fungal growth rate of *T. mentagrophytes* dermatophyte (MIC = 0.04 mg·mL^−1^).

Moreover, the results showed that the CNN essential oil was a little bit more effective on *M*. *gypseum* when compared to CNG_1_ and CNG_2_ samples (MIC equal to 0.2 vs. 0.4 mg·mL^−1^, respectively). On the other hand, CNG_2_ essential oil was less effective on the yeast *C*. *albicans*, with a MIC value of 0.4 mg·mL^−1^. 

### 2.3. Insecticidal Activity of the Test Oils 

The repellent properties of the three *C. nepeta* essential oils against *T. confusum* and *S. zeamais* adults were tested using the McDonald method. Table 4 gives the average repellency values of the essential oils tested at 2% concentration and for different exposure times.

We noticed that the repellent activity increased with a prolonged exposure time and varied depending on the tested sample. Thus, both populations of *Clinopodium nepeta* subsp. *glandulosum* (CNG_1_ and CNG_2_) had a prominent repellency effect against both insects (PR > 80%, after 2 h), while *C. nepeta* subsp. *nepeta* displayed a significantly low activity (PR < 60%, after 2 h), (*F_df2,11_* = 11.3; *p* = 0.003 for *T. confusum* and *F_df2,11_* = 23.5; *p* < 0.00 for *S. zeamais*).

Data presented in Table 4 showed that the tested oils exhibited, at 5% concentration, various degrees of toxicity against *T. confusum* and *S. zeamais* after 24 h exposure.

Using topical application bioassay, CNG_1_ and CNG_2_ essential oils were highly toxic to *S. zeamais*, inducing a mortality rate ranging from 97.5% for CNG_2_ to 100% for CNG_1_, respectively. However, they were weakly toxic to *T. confusum* (17.5% for both essential oils) (*F_df2,11_* = 8.16; *p* = 0.009).

On the other hand, the CNN essential oil induced moderate toxicity towards both adult species, with mortality rate values of 35% and 32.5% on *T. confusum* and *S. zeamais*, respectively (*F_df__2,11_* = 351.5; *p* < 0.001). 

## 3. Discussion

The main purpose of this study was to investigate for the first time the phytochemical variability in Tunisian *C. nepeta* essential oils taking into consideration the subspecies *nepeta* and *glandulosum* and their environmental origin (Béni-M’tir and Bizerta), and consequently its effect on the biological properties. As shown in Table 1, *C. nepeta* subsp. *nepeta* and subsp. *glandulosum* essential oils were mostly characterized by high contents of piperitone oxide and piperitenone oxide. Though the type oil resemblance between the subspecies, some quantitative and qualitative differences have been highlighted. Importantly, piperitone oxide which ranks first in CNN essential oil (51.7%), was identified as the second most abundant phytochemical (23.5%) in CNG_1_ after piperitenone oxide (39.3%). Nonetheless, the monoterpene piperitone ranks second in frequency in CNG_2_ population (19.5%) and was totally absent in the remaining analyzed samples. Some reports stated a strong relationship between the variability of *C. nepeta* essential oil chemical compositions and the geographical origin, environmental conditions and the vegetative state of the plants [31,35,36].

Thus, the observed variability in essential oil compositions is probably due to the various intrinsic genetic factors between the subspecies and different environmental aspects such as altitude and climate for both *glandulosum* subspecies collected in Bizerta and Béni-M’tir [37].

The literature data emphasized a great intraspecific variability of the natural volatile constituents of *C. nepeta* subsp. *nepeta* and subsp. *glandulosum* [30,32]. However, according to some authors, the chemical composition is independent of the subspecies *nepeta* or *glandulosum* as both constitute common sources of terpenoids and may produce the same major volatiles with the C-3 oxygenated *p*-menthane skeleton [33,38]. Others demonstrated a taxonomic understanding of the subspecies [39].

Some interesting observations were made on the diversity of the secondary metabolism of *C. nepeta* essential oils. Almost three types of volatile oils can be distinguished with some exceptions [40]. The first and most popular one consists mainly of pulegone associated with other cyclohexanones as menthone and piperitenone, along with piperitone oxide and piperitenoneoxide. The second kind of essential oil obtained from *Clinopodium* taxa is characterized by the predominance of piperitenone and/or of piperitone oxides, with the last one correlated with the presence of *iso*-pulegone and 1,8-cineole [32].

Thus, it is interesting to note that the essential oils of *C. nepeta* subsp. *nepeta* and subsp. *glandulosum* growing in Tunisia belong to the second type reported in literature as they were rich in piperitone oxide and piperitenone oxide. The compositions of the studied samples are almost similar to those of *C. nepeta* collected in Corsica [21].

Due to their safety characteristics and various aromatherapeutic effects, essential oils have got a lot of attention in several fields of modern chemistry to treat patients and as environmentally friendly preservatives. In this regard, we evaluated the antifungal activities of the aforementioned essential oils, taking into consideration the effect of the chemical polymorphism on the pharmacological effects of essential oils. 

Using the micro-dilution assay, the screening revealed that the antifungal effectiveness of all tested oils differs depending on the chemical composition as well as the dissimilarity of target fungus.

The most important effect was recorded by CNG_1_ essential oil on *T. mentagrophytes* (MIC = 0.04 mg/mL). In contrast, the CNG_2_ oil was less effective on this dermatophyte (MIC = 0.4 mg/mL), along with *A. flavus* (MIC > 2 mg/mL) and *C. albicans* (MIC = 0.4 mg/mL).

The differences may be related to the diversity of their chemical composition and the good antifungal effect of CNG_1_ essential oil against *T. mentagrophytes* may be due to the high amount of piperitenone oxide and piperitone oxide. Moreover, we suggest that the lower action of CNG_2_ oil against the same dermatophyte is due to the antagonist effect of piperitone quantified at a percentage of 19.5% in this volatile oil.

In the present study, *A. flavus* was the most resistant for all tested oils. Similarly and accordingly with previous reports, this mold presented the highest MIC value for *C. nepeta* subsp. *nepeta* essential oil from Portugal (MIC = 10 μL·mL^−1^) [31].

Moreover, CNN essential oil appeared to be the most effective on the dermatophyte *M. gypseum* showing the presence of compounds with known antifungal activity as the highly detected piperitone oxide (51.7%).

Similarly, previous studies confirmed the high antifungal activity of *C. nepeta* subsp. *nepeta* essential oil, especially against *Aspergillus* and dermatophyte molds [31]. After screening, the Italian *C. nepeta* volatile oil gave significant MIC values (0.32–1.25 μL·mL^−1^) in comparison with the less effective oils extracted in Portugal.

Herbivorous insects constitute the most exciting targets that facilitate chemical communication and adaptation to the environment. Repellents are substances with an offensive smell or taste, produced to fight off arthropod insect and to prevent attack from phytophagous. The uses of aromatic herbs and essential oils as insect repellents, has a long history in the herbal folklore [41].

This study represents the first report on the insecticidal efficacy of *C. nepeta* subspecies against *T. confusum* and *S. zeamais.*

The results showed that the repellent action was dependent upon the subspecies *nepeta* or *glandulosum,* while no significant differences were detected between the ability of both samples of *glandulosum* subspecies collected in Bizerta and Béni-M’tir regions to repel or kill the insects. Using the McDonald method, both essential oils at a concentration of 2% showed almost the same repellent activity against *T. confusum* and *S. zeamais* adults after 2 h of exposure. The percentages of repellency were 95 and 87.5% for CNG_1_ and CNG_2_, respectively, on *T. confusum* and 92.5% for both essential oils on *S. zeamais*. These oils fall under repellency class V (Highly Repulsive) according to Jilani and Su. [42]. However, 57.5% repellency of *C. nepeta* subsp. *nepeta* essential oil was observed against both adult insects, thus the volatile oil falls under class III repellency (Moderately Repulsive). 

Our results are in line with the repellent efficacy of *C. nepeta* essential oil towards *Aedes aegypti* mosquito. For instance, the essential oil gave promising scores for both space repellent properties and olfactory studies carried out on human volunteers [43].

Using the topical application bioassay, the application of these essential oils, at a concentration of 5%, resulted in mortality of *T. confusum* and *S. zeamais* within 24 h of exposure.

CNN essential oil was moderately toxic against both *T. confusum* and *S. zeamais*, whereas CNG_1_ and CNG_2_ essential oils were extremely toxic against *S. zeamais.* The mortality rate was 100 and 97.5%, respectively, after 24 h of exposure. For *T. confusum,* the mortality rate caused by *glandulosum* subspecies was 17.5%. Thus, the CNG_1_ and CNG_2_ essential oils presented a weaker activity against *T. castaneum* compared to the oil from the Montenegro accession of *C. nepeta* subsp. *glandulosum*, which showed mortality rates of 56.7 and 96.7% after 24 and 96 h of treatment, respectively. [27].

Previous literature data attributed the insecticidal properties of many essential oils to monoterpenoids, mainly the oxygenated ones, which are typically volatile compounds that can penetrate rapidly into the insects and interfere with their physiological functions [41,44]. 

Actually, the antifungal, repellent and insecticidal assays put in evidence that CNG_1_ essential oil was the most active one. This oil was characterized by the highest percentages of piperitenone oxide (39.3 vs. 23.4 and 27.8% of CNN and CNG_2,_ respectively). This monoterpenoid, endowed with an epoxide group, is typical of several *Mentha* L. essential oils, for instance *M. longifolia* L., *M. suaveolens* Ehrh. and *M. microphylla* K. Koch, which have demonstrated significant toxicity against stored product insects and fungal species [45,46,47]. Its biological power is given by the epoxide ring, which is interacting with proteins, neurotransmitters and nucleic acids [48]. Furthermore, piperitone oxide has been reported as a toxic repellent and reproduction retardant secondary metabolite towards the malarial vector *Anopheles stephensi* [49] as well as an effective antimicrobial agent [45]. In the present study, the *C. nepeta* subspecies oils showed differences in the activity against *T. confusum* and *S. zeamais*. Thus, repellent and toxic effects of the tested essential oils depend on the chemical composition variability as well as the insect susceptibility.

## 4. Materials and Methods 

### 4.1. Plant Material and Essential Oils Distillation 

The aerial parts of well-selected individuals from two native *C. nepeta* subspecies, i.e., subsp. *nepeta* and subsp. *glandulosum*, were harvested during the flowering stages from different regions/localities in North and North-Western of Tunisia (Table 1). Botanical identification of uninfected plant materials was authenticated by Dr. Ridha El Mokni, affiliated to the Department of Pharmaceutical Sciences “A”, Laboratory of Botany, Cryptogamy and Plant Biology, Faculty of Pharmacy of Monastir, Tunisia, where the voucher specimens have been preserved (Table 1). A quantity of 100 g of each sample was subjected to hydrodistillation in a glass Clevenger-type apparatus for 3 h. The oily fraction obtained on top of the aqueous phase at the end of each extraction was separated, dried over anhydrous sodium sulfate, filtered, and stored in the refrigerator until further analysis. The extraction yields were estimated on a dry weight basis (*w*/*w*).

### 4.2. Gas Chromatography–Mass Spectrometry (GC–MS)

Analyses of essential oil chemical compositions were performed using an Agilent 7890B gas chromatograph equipped with an auto sampler (PAL RSI 85) and coupled to a 5977B single quadrupole mass analyzer (Santa Clara, Californy, USA). Injection of 1 μL of the diluted sample (1:2000 dilution) in *n*-hexane (Carlo Erba, Milan, Italy) in the front inlet set at 280 °C was performed in split mode (1:100) with a split flow of 120 mL/min using an Agilent 5190-3983 liner (800 μL). Separation was performed using a 30 m × 0.25 mm i.d. × 0.25 μm film thickness, 5% phenylmethylpolysiloxane, HP-5MS capillary column (Agilent, Folsom, CA, USA) and helium (99.99%) was the carrier gas flown at 1.2 mL/min. The following oven temperature program was used: 60 °C for 5 min, then 4 °C/min up to 160 °C, then 11 °C/min up to 280 °C with a hold time of 15 min, and finally 15 °C/min until 300 °C for a total run time of 57.74 min. MSD transfer line temperature was set at 300 °C. Analysis was made in electron impact (EI) mode (internal ionization source; 70 eV) with a scan range from 29 to 400 m/z, after a solvent delay of 2.5 min. The compounds were identified by two approaches: (i) correspondence of retention indices (RIs) reported in libraries [50] (NIST 17, 2017; FFNSC 2, 2012) with the ones calculated using a mixture of n-alkanes (C_8_–C_30_, Supelco, Bellefonte, CA, USA); (ii) comparison of the obtained mass spectra with those stored in libraries (WILEY275, ADAMS, NIST 17 and FFNSC 2); (iii) co-injection with available analytical standards. The chromatograms have been integrated and the relative abundance (%) of each compound was obtained (% = 100 × peakarea/totalpeakarea). The repeatability is expressed by coefficient of variation (CV)% obtained performing the GC/MS analyses in triplicate of the different samples. The coefficient of variation obtained ranged from 0.3% to 5.8%. The inter-day repeatability of the GC/MS method was determined by 3-day replicate analyses of volatiles, evaluated on the same aliquot of sample stored in the refrigerator, CV% ranged from 0.1% to 3.9%, accounting for very high constant results.

### 4.3. Antifungal Activity

#### 4.3.1. Fungal Isolates 

The antifungal effectiveness of *C. nepeta* subspecies essential oils was tested against the animal source dermatophytes: *Microsporum canis*, *Microsporum gypseum* and *Trichophyton mentagrophytes,* the environmental origin molds: *Aspergillus flavus*, *Aspergillus terreus* and the yeast *Candida albicans.*

#### 4.3.2. Microdilution Test 

The antifungal susceptibility tests were carried out using a microdilution assay, according to the Clinical and Laboratory Standards Institute (CLSI) M38A_2_ recommendations for molds [51], and those of CLSI M27A_3_ for yeasts [52]. Essential oils were assayed at different concentrations (2, 1.8, 1.6, 1.4, 1.2, 1, 0.8, 0.6, 0.4, 0.2, 0.08, 0.06, 0.04, and 0.02 mg/mL). All procedures were performed in triplicate.

### 4.4. Insecticidal Activity 

#### 4.4.1. Tested Insects and Rearing Conditions

*Tribolium confusum* and *Sitophilus zeamais* were taken from the Laboratory of Entomology, Regional Research Center on Horticulture and Organic Agriculture, Chott-Mariem (CRRHAB), Tunisia. Insect adults were cultured in a growth cabinet set at the following rearing conditions: 28 ± 1 °C, 60% relative humidity (RI), 16 h light and 8 h dark photoperiod, without exposure to any insecticidal contamination. The food media used were wheat flour for *T*. *confusum* and whole maize grains for *S*. *zeamais*.

#### 4.4.2. Repellent Activity

The repellency test against *T. confusum* and *S. zeamais* beetle adults was assessed following McDonald et al. [53] method. Briefly, 200 μL of each essential oil solution, adjusted at a concentration of 2.0%, were applied to a half Whatman filter paper (No.1) disc of 9 cm diameter. The other half, used as a control, was steeped with 200 μL of pure acetone. After air-drying for 10 min, treated and untreated halves were attached together. Then, 20 adult insects of both species were released separately at the center of the filter paper disc then placed into Petri dishes. After 15, 30, 60, and 120 min from the beginning of the assay the numbers of insects present on the control (Nc) and on the treated (Nt) areas were registered. Each experiment was performed in four repetitions. The repellency percentage values (PR) were computed as follows:

PR=[(Nc−Nt)/(Nc+Nt)]×100

The resulting values were used for the classification of essential oils in different repellency classes suggested by Jilani and Su [42].

#### 4.4.3. Contact Toxicity: Topical Application Bioassay

*C. nepeta* subspecies essential oils were tested against *T. confusum* and *S. zeamais* following the method of Liu and Ho [54]. Aliquots of 1 µL from each sample at 5% concentration (10 μL of each EO dissolved in acetone) were topically applied on the thorax of insect adults using a micropipette. Insect controls were treated only with acetone. After evaporating the solvent, groups of 10 adults were introduced on glass Petri dishes (9 cm diameter). Four repetitions were carried out for each experiment. Petri dishes were kept under the same rearing conditions described above and insects mortality was recorded after 24 h of treatment (until the number of dead insects stabilized). Insects that did not record any movements were considered as dead. Abbott’s formula [55] was used to correct the mortality rate:

%Mc=[(M0−Mt)/(100−Mt)]×100

where Mc: Corrected mortality rate; and M_0_ and Mt: Mortality rate of treated and control insects, respectively. 

#### 4.4.4. Data Analysis

Statistical analyses were performed using ANOVA followed by Tukey’HSD test. SPSS 20 software was used to perform all tests.

## 5. Conclusions

The obtained results showed that *Clinopodium nepeta* subsp. *nepeta* and *C. nepeta* subsp. *glandulosum* essential oils presented chemical variability depending on the subspecies and the geographical location of plant materials. The monoterpenoid-rich essential oils demonstrated high antifungal activities against dermatophytes, molds and yeasts with different efficiencies. Given the pronounced repellent and toxic effects towards *T. confusum* and *S. zeamais*, *C. nepeta* subsp. *glandulosum* oils may be considered as promising candidates to control those insect pests during storage. To better understand the pharmacological effects of the analyzed samples, further investigation on the effective major compounds will be carried out. Added to that, an exploration of the synergistic interactions, the antagonist effects and the environmental safety is imperative before suggesting them as fungal drugs or safe alternatives to grain protectants.

## Figures and Tables

**Table 1:** Locality/Harvesting place, harvesting period and voucher specimen reference of both *Clinopodium nepeta* subspecies.

Taxon	Species Abbreviation	Harvesting Place	Harvesting Period (2016)	Voucher Specimen
*Clinopodium nepeta* (L.) Kuntze subsp. *nepeta*	CNN	Béni-M’tir	October	[LAM./Cal.n.n./Kroumiria/BM.13/27102016]
*Clinopodium nepeta* subsp. *glandulosum* (Req.) Govaerts	CNG_1_	Béni-M’tir	October	[LAM./Cal.n.g./Kroumiria/BM.25/27102016]
*Clinopodium nepeta* subsp. *glandulosum* (Req.) Govaerts	CNG_2_	Bizerte	July	[LAM./Cal.n.g./NE/Bizerta. 03/10082016]

**Table 2:** Chemical profiles of essential oils obtained from *Clinopodium nepeta* subspecies harvested from different localities.

No.	RI Calc	RI LIT	Compounds	Content (%)		
				CNN	CNG_1_	CNG_2_
1	932	932	α-Pinene	0.23	0.2	0.1
2	975	974	β-Pinene	0.3	0.3	0.1
3	991	988	Myrcene	-	0.1	tr
4	995	994	3-Octanol	0.6	0.7	0.7
5	1000	1000	Decane	tr	-	
6	1024	1024	*o*-Cymene			tr
7	1024	1022	*p*-Cymene	-	0.3	-
8	1028	1024	Limonene	1.9	4.2	1.4
9	1030	1026	1,8-cineole	0.4	0.2	0.1
10	1058	1054	γ-Terpinene	tr	0.2	tr
11	1066	1070	*cis*-4-Thujanol	-	0.1	-
12	1100	1095	Linalool	0.7	0.6	0.5
13	1120	1119	*trans*-*p*-Mentha-2,8-dien-1-ol	-	0.1	tr
14	1164	1165	Borneol	-	0.3	0.4
15	1176	1174	Terpinen-4-ol	-	1.1	0.1
16	1184	1179	*p*-Cymen-8-ol	-	0.6	0.2
17	1189	1186	α-Terpineol	0.4	0.4	0.4
18	1197	1196	Methyl chavicol	-	0.3	-
19	1211	1220	4,7-dimethylbenzofuran	-	0.2	tr
20	1215	1221	8,9-Dehydrothymol	-	0.3	0.4
21	1239	1238	Cumin aldehyde	-	2.0	0.1
22	1243	1239	Carvone	-	0.2	tr
23	1253	1249	Piperitone	-	-	19.5
24	1255	1253	Piperitone oxide	51.7	23.5	16.3
25	1268	1274	Pseudodiosphenol	-	-	0.2
26	1271	1277 ^a^	*p*-Mentha-1,8-dien-3-one	-	0.5	0.6
27	1286	1287	Bornyl acetate	0.3	0.3	0.2
28	1289	1298	*p*-Mentha-1,4-dien-7-al	-	1.0	0.2
29	1292	1289	Thymol	3.6	1.6	4.0
30	1299	1305	Diosphenol	0.6	-	1.1
31	1302	1308	6-Hydroxycarvotanacetone	0.7	5.1	1.2
32	1340	1340	Piperitenone	0.2	0.4	0.5
33	1366	1366	Piperitenone oxide	23.4	39.3	27.8
34	1376	1374	α-Copaene	-	0.6	0.3
35	1385	1387	β-Bourbonene	-	0.4	0.2
36	1400	1400	Tetradecane	0.2	0.9	1.8
37	1419	1417	(*E*)-Caryophyllene	0.3	1.4	0.6
38	1454	1452	α-Humulene	tr	0.1	tr
39	1458	1454	(*E*)-β-Farnesene	-	0.1	tr
40	1481	1484	Germacrene D	-	0.3	tr
41	1524	1522	δ-Cadinene	-	0.2	tr
42	1578	1577	Spathulenol	tr	-	0.2
43	1583	1582	Caryophyllene oxide	2.0	2.7	2.3
44	1600	1600	Hexadecane	-	tr	-
45	1613	1608	Humulene epoxide II	tr	0.2	0.2
46	1689	1687	Eudesma-4(15),7-dien-1β-ol	-	0.3	0.2
47	1848	1844	Phytone	tr	-	tr
Oxygenated monoterpenes				82.0	77.6	73.4
Monoterpene hydrocarbons				2.5	5.3	2.3
Oxygenated sesquiterpenes				2.1	3.1	2.8
Sesquiterpene hydrocarbons				0.4	3.1	1.2
Others				1.0	2.1	2.7
Total identified components				88.0	91.2	82.4

RI Calc: linear retention index calculated against homologue series of C_8_–C_30_ alkanes. RI LIT: RI taken from Adams (2007) or NIST 17 (2017). Tr: Traces, % < 0.1. ^a^ RI value taken from [34].

**Table 3:** Antifungal properties of essential oils produced by hydrodistillation of *Clinopodium nepeta* subsp. *nepeta* and *C. nepeta* subsp. *glandulosum* (MIC, mg·mL^−1^).

Fungal Strains	Essential Oils		
	CNN	CNG_1_	CNG_2_
*Aspergillus flavus*	2	2	>2
*Aspergillus terreus*	0.4	0.4	0.4
*Candida albicans*	0.2	0.2	0.4
*Microsporum canis*	0.4	0.4	0.4
*Microsporum gypseum*	0.2	0.4	0.4
*Trichophyton mentagrophytes*	0.2	0.04	0.4

**Table 4:** Repellency and toxicity of *Tribolium confusum* and *Sitophilus zeamais* exposed to the *C. nepeta subspecies* essential oils (2% and 5% concentrations for repellency and mortality tests, respectively) [A].

Essential Oils Plant Source	Exposure Duration (min)	Repellency (%)		Mortality (%)	
		*T. confusum*	*S. zeamais*	*T. confusum*	*S. zeamais*
CNN	15	52.5 ± 9.57 ^a^	22.5 ± 9.57 ^a^		
	30	52.5 ± 5.00 ^a^	40 ± 8.16 ^a^		
	60	55 ± 5.77 ^a^	57.5 ± 9.57 ^a^	35 ± 5.00 ^b^	32.5 ± 5.00 ^a^
	120	57.5 ± 18.92 ^a^	57.5 ± 9.57 ^a^		
CNG_1_	15	82.5 ± 9.57 ^b^	60 ± 11.54 ^b^		
	30	85 ± 10.00 ^b^	62.5 ± 9.57 ^b^	17.5 ± 8.00 ^a^	100 ± 0.00 ^b^
	60	92.5 ± 5.00 ^b^	92.5 ± 9.57 ^b^		
	120	95 ± 5.77 ^b^	92.5 ± 5.00 ^b^		
CNG_2_	15	80 ± 18.25 ^b^	87.5 ± 18.92 ^b^		
	30	80 ± 8.16 ^b^	87.5 ± 5.00 ^c^	17.5 ± 5.00 ^a^	97.5 ± 5.00 ^b^
	60	82.5 ± 9.57 ^b^	90 ± 0.00 ^b^		
	120	87.5 ± 5.00 ^b^	92.5 ± 9.57 ^b^		

[A] Data are mean ± SE (*n* = 4). Means with same alphabetic letters are not significantly different at *p* < 0.01 using Tukey’s HSD test between essential oil plant sources for the same exposure duration and same insect species.
